# Complete genome sequence of *Kitasatospora aureofaciens* Tü117

**DOI:** 10.1128/MRA.01014-23

**Published:** 2024-01-17

**Authors:** Shotaro Kato, Satoshi Yuzawa, Tomoki Takeda, Kazuharu Arakawa

**Affiliations:** 1 Institute for Advanced Biosciences, Keio University, Tsuruoka, Yamagata, Japan; 2 Systems Biology Program, Graduate School of Media and Governance, Keio University, Fujisawa, Kanagawa, Japan; 3 Faculty of Environment and Information Studies, Keio University, Fujisawa, Kanagawa, Japan; University of Strathclyde, Glasgow, UK

**Keywords:** *Kitasatospora aureofaciens*, complete genome, nanopore sequencing, genome assembly, α-lipomycin

## Abstract

*Actinobacteria* produce about two-thirds of all naturally derived antibiotics currently in clinical use. *Kitasatospora aureofaciens* Tü117 is a species of *Actinobacteria* and produces α-lipomycin. We report the complete genome sequence of *K. aureofaciens*, composed of a single linear chromosome of 8,717,539 Mbp with a G + C content of 72.0%.

## ANNOUNCEMENT

*Actinobacteria* are active producers of secondary metabolites, producing approximately two-thirds of all naturally derived antibiotics currently in clinical use (1). *Kitasatospora aureofaciens* is a species of *Actinobacteria* and produces α-lipomycin that is an orange-red acyclic polyene antibiotic (1). This was named lipomycin because the antibiotic activity is antagonized by several lipids, including lecithin and some sterols (2). The genome sequence of *K. aureofaciens* Tü117, the producer of α-lipomycin (3), may be helpful for the generation of novel natural products, including antibiotics. To this end, we report the complete genome sequence of *K. aureofaciens* Tü117.

*K. aureofaciens* Tü117 was originally isolated from soil sample from Venezuela (3), and its glycerol stock was obtained from Andreas Bechthold (Department of Pharmaceutical Biology and Biotechnology, Institute of Pharmaceutical Sciences, Albert-Ludwig University of Freiburg). A 2% inoculum was cultured for 2 days under aerobic conditions at 30°C in an actinomycete incubator Bioshaker (BR-21FP; TAITEC, Koshigaya, Japan) in Tryptic Soy Broth liquid medium with 0.1% nalidixic acid. Genomic DNA was purified using Genomic-tip 20/G (QIAGEN, Hilden, Germany), and a single extract was used for both of the long- and short-read sequencing. Long-read sequencing libraries were prepared and multiplexed using the Rapid Barcoding Kit (SQK-RBK004; Oxford Nanopore Technologies, Oxford, UK). No size selection was performed prior to nanopore sequencing. Libraries were sequenced with FR10.3 Flowcell (FLO-MIN111), base called with Guppy version 6.3.8 (Super Accurate Mode), demultiplexed, and adapter-trimmed on the GridION X5 device (GridION software release 21.10.5; Oxford Nanopore Technologies, Oxford, UK). The quality of long reads was checked using Nanoplot version 1.33.0 (4), resulting in a total of 274.8 Mbp consisting of 69,859 reads of N50 length 7,789  bp. Reads longer than 10 kb were used to assemble using Canu version 2.2 (5). The resulting single contig was manually confirmed to be a full-length linear chromosome like other *Streptomyces* genomes, by whole genome alignment with closely related species *Streptomyces pactum* ACT12 (CP019724.1) with D-Genies server (accessed on 12 December 2023) (6), as well as by online NCBI BLAST searches (blastn; accessed on 12 December 2023) of 10-kb terminal sequences, where the position 1..10,000 matched the left-most terminal sequence of *Streptomyces pactum* ACT12 (CP019724.1) genome with 98% identity and 86% query coverage, and the position 8,716,540.. 8,717,539 matched the right-most terminal sequence of *Streptomyces violaceoruber* CGMCC 4.1801 (CP137734.1) with 91% identity and 42% query coverage. An Illumina sequencing library was prepared using a KAPA HyperPlus kit (Kapa Biosystems, Cape Town, South Africa) for error correction, and the library was sequenced on a MiSeq sequencer (Illumina, San Diego, USA) using the Reagent Kit v3 600 cycles as 300 bp paired-end reads. Unfiltered Illumina short reads (5.5 M pairs) were used for error correction using Pilon version 1.2.3 for three rounds (7). The assembly was checked for completeness using CheckM 1.2.2 (8) to be 100% completeness and 0.98% possible contamination rate using automatic lineage model selection. The assembly was then annotated using DDBJ Fast Annotation and Submission Tool (DFAST) version 1.2.18 (9). Default parameters were used except where otherwise noted.

The annotated linear genome of *K. aureofaciens* is 8,717,539 bp with a G + C content of 72.0%, containing 7,956 putative coding sequences, 18 rRNA genes, 80 tRNA genes, and 0 CRISPR loci were predicted, at 31.52 × coverage.

## Data Availability

The genome sequences reported here were deposited in DDBJ under accession number AP028963, and the raw reads were deposited in the Sequence Read Archive (SRA) under BioProject accession number PRJNA1012901 as SRR25906302 and SRR25906303 runs.
